# Trusting health information from large language models, online articles, and discussion forums: A comparison of perceived credibility

**DOI:** 10.1016/j.invent.2026.101015

**Published:** 2026-11-06

**Authors:** Katarzyna Zalass-Knyziak, Katarzyna Cantarero

**Affiliations:** a Faculty of Psychology in Wroclaw, SWPS University, Poland; b School of Psychological Science, University of Bristol, United Kingdom

**Keywords:** Credibility, Health (mis)information, Presentation format, Disinformation, AI, Large language model

## Abstract

While the increasing use of Large Language Models (LLMs) as eHealth tools facilitates rapid information retrieval, it also carries the risk of credibility inflation for medically inaccurate content. The aim of the study was to examine the effects of the form of presentation of health information (LLM response vs. online news article vs. online forum) and its truthfulness on perceived credibility judgments. The study (*N* = 405) employed a mixed-design experiment, with presentation format as a between-subjects factor and true vs false health information as a within-subjects factor. The results showed that although participants were generally able to distinguish between true and false information, the form of presentation significantly moderated this process with a medium effect size (*η*^*2*^_*p*_ = 0.07). Specifically, both LLM and online forum elevated credibility rating of false medical information. The findings have important implications for understanding cognitive mechanisms underlying credibility judgments in digital environments and for the design of safe and responsible eHealth tools.

## Highlights

• Presentation format shaped credibility of online health information.
• True health information was rated as more credible than false information.
• False health information seemed more credible as LLM and forum content.
• Frequency of LLM use did not predict credibility ratings of AI-generated content.
• Article format best supported differentiation between true and false health information.

The contemporary digital information ecosystem is profoundly challenged by the proliferation of false and misleading information, a global phenomenon that threatens both democratic legitimacy and public health (e.g., Lewandowsky et al., 2017). Concern often centers on social media, yet search engines occupy an equally consequential but less examined position as gatekeepers of what people encounter when they set out to verify a claim. This deserves attention because searching online to verify a news story can paradoxically increase belief in false content, mainly because of reliance on lower-quality sources (Aslett et al., 2024). Subsequent work extends this showing that what matters is not the act of searching but how people search: queries that reuse the vocabulary of the source article predict both lower-quality results and greater trust in false news (Elsweiler et al., 2025). These mechanisms have been observed for both political and health-related content (Aslett et al., 2024), which brings the problem directly into the health domain.

Within this broader context of digital uncertainty, the eHealth domain is particularly sensitive. A large majority of adults use the Web to search for health information, making medical queries a major share of search engine traffic (White and Horvitz, 2009). Although eHealth offers many advantages, it also presents considerable challenges (Whitten et al., 2001). For example, using web search to evaluate symptoms or medical claims can lead to an unfounded escalation of concern, often termed cyberchondria (White and Horvitz, 2009). This is perhaps unsurprising, given that the health domain is characterized by high uncertainty, elevated anxiety, and limited opportunities for lay users to verify information substantively (e.g., Luo et al., 2026; Peng, 2022).

Discerning between true health information and medical misinformation is a challenge that patients face (Daraz et al., 2019). In the health domain, during the diagnostic process, users may often experience a heightened state of anxiety, which substantially taxes cognitive resources. In terms of the Elaboration Likelihood Model (ELM) (Petty and Cacioppo, 1986), the situation of high personal relevance combined with low ability to evaluate content on its merits pushes individuals toward the peripheral route of processing. Consequently, it can foster a drive for rapid cognitive closure (Kruglanski and Webster, 1996), evaluating eHealth information heuristically (Tang et al., 2026) and health information fatigue (Luo et al., 2026), at the expense of careful information verification.

In such circumstances, technology may begin to function not only as an informational tool but also as a kind of cognitive offloading tool in that it is an external resource that substitutes internal cognitive efforts (Risko and Gilbert, 2016). Saunders (2024) suggests that, faced with the complexity of the contemporary world, users may be inclined to trust machines precisely because they appear to offer unambiguous answers and reduce uncertainty more effectively than nuanced, cautious human messages. In the context of the present study, this leads to the assumption that language models (such as ChatGPT) due to their immediacy, categorical tone, and high clarity, may satisfy the need to reduce anxiety more effectively than traditional medical texts.

## 1 Cognitive mechanisms of truth verification: automatic acceptance and cognitive control

The evaluation of information credibility is not a single, uniform decision, but a complex cognitive process that unfolds in stages (Metzger, 2007; Wathen and Burkell, 2002). Several theoretical frameworks inform our understanding of how the human mind evaluates claims about the world in terms of their truthfulness.

### 1.1 The Spinozan model of information processing

A key account for explaining these mechanisms is the theory proposed by Gilbert et al. (1990), which draws on the philosophical debate between Descartes and Spinoza. The Cartesian view assumes that the mind first registers information and only subsequently evaluates it logically, deciding whether to accept or reject it. Based on experimental findings, Gilbert et al. (1990) proposed an alternative account commonly referred to as the Spinozan model.

According to this approach, the act of understanding a message is psychologically equivalent to accepting it. Belief in the content arises automatically, whereas verification and, if needed, the “unbelieving” of false information, becomes possible only at a later, second stage. This stage, however, requires time, cognitive resources, and active attentional control.

Under conditions of cognitive load, stress, or time pressure, which are characteristic of internet use and online health information seeking, this process may be disrupted or prematurely terminated. As a result, recipients may remain at the initial stage of automatic acceptance, treating the message as true even when the information it contains is inconsistent with the facts (Gilbert et al., 1990).

### 1.2 Truth judgments

An extension of the concept of automatic acceptance is Levine's (2014) Truth-Default Theory (TDT). According to this account, humans are evolutionarily predisposed to assume honesty in communication. Recipients treat messages as true by default unless a clear cue (a “trigger”) emerges that suggests the possibility of deception or manipulation. This mechanism serves an adaptive function in direct social relationships, enabling efficient functioning in an environment grounded in cooperation and trust. However, judgments of truth do not always align with the ground truth of the events. For instance, the *illusory truth effect* suggests that repeated exposure to information, regardless of its factual accuracy, increases its perceived validity by enhancing processing fluency (Hasher et al., 1977; Fazio et al., 2015).

In digital environments, however, the assumption of truth by default may be modified. Contemporary misinformation research points to a phenomenon known as skepticism bias, which appears to be characteristic of digital media settings (Pfänder and Altay, 2025). Contrary to common concerns about widespread gullibility among internet users, numerous empirical analyses, including a meta-analysis involving more than 190,000 participants, show that recipients more often make the error of rejecting true information (including information supported by scientific evidence) than of uncritically accepting falsehoods (Pfänder and Altay, 2025).

Pfänder and Altay (2025) showed that users' ability to distinguish between true and false information remains relatively high. At the same time, overall trust in digital media is reduced, which shifts the decision criterion toward excessive caution. As a result, recipients are more likely to respond with skepticism toward messages as such, regardless of their actual epistemic value.

In the context of health information, this mechanism may lead to a paradoxical situation in which a patient acting out of caution rejects reliable medical knowledge, treating it defensively as potential misinformation. This has important implications for interpreting credibility judgments, as it suggests that low credibility ratings do not always reflect accurate detection of falsehood but may instead stem from a general tendency toward skepticism about online sources.

## 2 Credibility judgments in digital media

Credibility is not an objective property of information; rather, it is a subjective judgment formed by the recipient during message processing. This implies that credibility assessment is not a simple reaction to informational content but an interpretive outcome in which both message characteristics and the context of its presentation play a central role (Appelman and Sundar, 2016; Kiousis, 2001; Winter and Krämer, 2014).

Message credibility focuses on characteristics of the content itself, regardless of who authored it. Appelman and Sundar (2016) define it as the recipient's subjective judgment of a message's truthfulness, based on the perceived quality and coherence of the message. Key indicators of message credibility include, among others, authenticity, accuracy, and the overall believability of the content (Appelman and Sundar, 2016).

Medium credibility is understood as the perceived credibility of the communication channel in which information is presented. Researchers such as Kiousis (2001) and Flanagin and Metzger (2000) argue that the medium itself, independent of the source and the content, may carry its own “baseline” credibility, shaped by recipients' prior experiences, cultural norms, and social associations linked to a given communication channel. In a recent study, AI-generated mental health responses were perceived as more authentic, professional and practical than human-generated when the source was not disclosed, but disclosing the source significantly lowered perceptions of authenticity in AI-generated assistance (Jain et al., 2024).

Recipients may therefore evaluate the same information differently depending on whether it is presented as a traditional online article, a post on a discussion forum, or a conversation with an AI-based chatbot. In such cases, the medium functions as an interpretive context that shapes credibility judgments even before the content is processed on its merits.

This means that the format in which health information is presented may function as an independent credibility cue, regardless of the content's actual truthfulness. The medium does not merely convey information; it actively shapes reception by influencing how users interpret and evaluate the message.

## 3 Psychological determinants of trust in algorithms: heuristics and cognitive biases

There are at least two opposing narratives about trust in algorithmic systems, both of which are central to interpreting findings on the credibility of AI-generated content. The first is based on the concept of algorithm aversion, which suggests that people are more reluctant to rely on automated systems than on humans, especially after observing an algorithm's error. Research indicates that algorithms are perceived as lacking the ability to understand contextual nuances, intentions, and moral considerations, which can reduce trust in the content they generate, particularly in socially sensitive domains such as health and ethics (Longoni et al., 2022).

On the other hand, a substantial body of empirical work documents algorithm appreciation, explained in part by the machine heuristic described within the MAIN model (Sundar, 2008). According to this view, users apply a cognitive shortcut whereby content generated by computer systems is perceived as more objective and free from human biases, emotions, and hidden intentions. Under certain conditions, the “AI” label may therefore function as a positive peripheral cue, increasing perceived message credibility.

Contemporary research on generative artificial intelligence, including large language models such as ChatGPT, suggests a third mechanism that may best explain how users actually evaluate the credibility of this type of content when directly engaging with the message. As Huschens et al. (2023) argue, the key factor may be not the source label itself (“AI” vs. “human”) but the message's formal quality. Texts generated by language models are characterized by high linguistic fluency, grammatical correctness, and a clear structure.

In the ELM (Petty and Cacioppo, 1986), these features constitute strong peripheral rather than central cues that can increase perceived credibility regardless of the content's substantive accuracy. As a result, users may not report differences in source credibility at the declarative level, yet in practice may judge AI-generated responses as more credible than comparable human-written content because they are easier to process cognitively and appear coherent and “confident” (Huschens et al., 2023). Given the documented difficulty of judging health misinformation as such (Daraz et al., 2024), both the formal quality mechanism and algorithm appreciation are likely to inflate credibility judgments of false claims presented in LLM-generated eHealth content.

## 4 Study aim and hypotheses

The aim of the present study was to examine how the format of health information presentation and the truthfulness of that information affect recipients' subjective evaluations of content credibility. Specifically, the study analyzed differences in credibility ratings for the same information when presented as an online article, a conversation with a language model, or a discussion forum post, as well as participants' ability to distinguish true from false information across presentation formats. We included a third condition, the discussion forum, given that online articles and LLM-generated content differ not only in the perceived source of the information, but also in format (i.e., conversational vs. plain text). While discussion forums share a conversational format with LLM-generated content, they differ in the perceived authorship and may provide a different form of reassurance, grounded in social proof and the shared experiences of other users.

Based on the literature review and the theoretical models presented above, the following research hypotheses were formulated:

H1 Texts presented as responses generated by a language model (LLM) are rated as more credible than the same texts presented in the format of an online article. Additionally, we include a discussion forum condition to control for the effects of conversational format independently of the AI source.

H2 True information is rated as more credible than false information, regardless of presentation format.

H3 Presentation format (article vs. LLM) interacts with information truthfulness: for false texts, the LLM format increases credibility ratings more than the news format.

In addition, the following exploratory hypothesis was preregistered:

H4 More frequent use of language models (LLMs) by participants is positively associated with higher credibility ratings for texts presented in the LLM-response format.

## 5 Method

The study was designed as an experiment using a 3 × 2 mixed factorial design. The first independent variable was information presentation format (a between-subjects factor) with three levels: (1) an online article, (2) a conversation with an AI assistant (ChatGPT), and (3) a discussion forum post. The second independent variable was information truthfulness (a within-subjects factor) with two levels: true information (consistent with current medical knowledge) and false information (inconsistent with medical knowledge). The dependent variable was the subjective credibility rating of the presented content. In addition, to reduce the likelihood of alternative explanations for potential between-condition differences stemming from varying levels of interactivity or engagement across formats, the materials in all conditions were standardized as closely as possible in terms of length, structure, and the scope of information provided.

The study design, including the hypotheses and the analysis plan, was preregistered on the AsPredicted platform and is available at: https://aspredicted.org/i73xb4.pdf. The data file and the materials are available at https://osf.io/t27sn.

### 5.1 Participants

Recruitment followed two parallel routes. Most records (*n* = 397; 69%) were obtained via the university SONA system, with students receiving course credit, whereas the remaining records (*n* = 177; 31%) came from individuals outside the academic setting (recruited through social media). In total, 574 survey entries were recorded.

To ensure high data quality, a multi-step sample selection procedure was applied. From the initial pool, participants were excluded if they did not complete the study procedure (*n* = 78), did not provide consent to participate (*n* = 9), responded incorrectly to the attention check (*n* = 33), or incorrectly identified the presentation format of the texts in the comprehension check (*n* = 49).

The final sample included *N* = 405 participants. The sample was predominantly female (*n* = 324; 80%), with men comprising 19% (*n* = 78), and 3 participants (1%) reporting another gender. Participants' ages ranged from 19 to 71 years, with a mean age of *M* = 28.60 years (*SD* = 10.50). In terms of education, the sample was dominated by participants with secondary education (*n* = 230; 57%) and higher education (*n* = 166; 41%). The remaining participants reported vocational education (*n* = 6; 2%) or primary education (*n* = 3; 1%).

### 5.2 Materials and experimental manipulation

The experimental materials consisted of a set of four health-related texts developed specifically for the present study. The four topics were selected from fact-checks published by the service FirstCheck.in between 2023 and 2025. Selection criteria were that the claim concerned everyday health behavior rather than clinical decision-making, that the service had issued an explicit verdict on its accuracy, and that the claim was not tied to a specific national health system or cultural practice, so that it would be interpretable outside the Polish context. The classification of each text as true or false followed the verdict issued by the fact-checking service: foot immersion in warm water as support in reducing fever (Richhariya, 2025) and the ineffectiveness of vitamin C supplementation in preventing infections (First Check, 2023) were classified as true, whereas auto-urine therapy (Vyas, 2025) and the claim that 80% of patients with hypertension do not require pharmacological treatment (First Check, 2025) were classified as false. It should be noted that the evidence base differs in strength across the true items: the ineffectiveness of vitamin C supplementation reflects broad scientific consensus, whereas the claim concerning foot immersion rests on more limited evidence. The set thus included two texts containing true information (regarding home-based methods for reducing fever and the lack of effectiveness of vitamin C in preventing infections) and two texts containing false information (promoting urine therapy and denying the need to treat hypertension). These contents constituted the levels of the within-subjects variable (information truthfulness). The materials were presented in Polish; English translations of all stimuli are provided in the Supplementary Materials. The original stimuli are available from the authors upon request.

### 5.3 Measures and variables

The primary dependent variable was the subjective credibility rating of the presented content. Credibility was assessed immediately after exposure to each text using a single item: “Rate the overall credibility of the information presented in the text.” Responses were given on a 5-point Likert-type scale (1 = *Definitely not credible*, 5 = *Definitely credible*), with the midpoint indicating a neutral position. Credibility was operationalized as message credibility, that is, the recipient's subjective judgment of the truthfulness of the content itself (Appelman and Sundar, 2016). A single-item format was chosen because the within-subjects design required participants to evaluate four stimuli in succession; single-item veracity and credibility judgments are standard in misinformation research for precisely this reason, as they capture immediate perceptual judgments while limiting fatigue across repeated trials (Aslett et al., 2024).

To characterize the sample and control for potential confounding factors, additional variables were assessed: (a) familiarity with and frequency of use of LLMs (scales ranging from “no familiarity”/“never” to “expert”/“daily”), (b) habits related to seeking health information online, and (c) prior familiarity with the presented topics (novelty ratings on

a 7-point scale). Novelty ratings were included because processing fluency arising from prior exposure is known to inflate perceived validity irrespective of factual accuracy, as described by the illusory truth effect (Hasher et al., 1977; Fazio et al., 2015). The remaining items concerning familiarity with language models and health information-seeking habits were developed for the present study and are reported in full in the Supplementary Materials.

### 5.4 Procedure

The study was conducted online using the Qualtrics platform. The procedure was automated. After reading information about the study aim, anonymity, and the estimated duration, participants provided informed consent. They then completed a sociodemographic questionnaire.

Next, the system randomly assigned each participant to one of three experimental conditions (Article, ChatGPT, or Forum). Within the assigned condition, participants were sequentially presented with four texts (two true and two false) in a randomized order. After each text, the dependent variable was assessed. After completing the main task, an item serving as an attention check was presented (*To ensure the high quality of the data collected, the study includes a question to verify careful reading of the instructions. Here is one such question. Regardless of what you think, please select “Rather credible”. Thank you for your understanding. It is important to ensure that the scientific data are reliable*. 1 = *Definitely not credible*, 5 = *Definitely credible*). Next, additional variables were assessed, and participants were asked whether they had recognized the format in which the texts had been presented, which was our comprehension check question (*The texts presented in the study were in the form of*: 1 = *An online article*, 2 = *A conversation with ChatGPT*, 3 = *A discussion forum post*). All the measured variables are reported at https://osf.io/t27sn.

#### 5.4.1 Ethical considerations

The study was designed and conducted in accordance with ethical principles for psychological research. Before starting the procedure, each participant was provided with information about the study aim and procedure, and participation required voluntary and informed consent. The introductory instructions clearly stated that participation was fully anonymous and that participants had the right to withdraw at any time without giving a reason and without any consequences.

Given the nature of the experiment, which required exposing participants to false medical information (health myths concerning, among other topics, hypertension treatment and urine therapy), the procedure included a full debriefing. Immediately after the measurement phase, participants were shown detailed feedback clearly indicating which of the presented claims were consistent with current medical knowledge and which were false. Participants also received corrections of the inaccurate statements (e.g., an explanation of the lack of effectiveness of urine therapy and the necessity of treating hypertension), in order to minimize the risk of reinforcing false health beliefs. At the end of the study, participants were also provided with the author's email address to enable contact in case of questions or concerns.

## 6 Results

The aim of the study was to examine whether the format of health information presentation (an online article, an AI conversation, or a discussion forum) and its truthfulness affect recipients' credibility ratings. In addition, the study tested the association between the frequency of using these sources and participants' self-reported decisions to forgo a medical visit.

Descriptive statistics for credibility ratings by experimental condition are presented in Table 1.

**Table 1:** Descriptive Statistics for Credibility Ratings by Presentation Format and Information Truthfulness.

Experimental condition	Text truthfulness	*M*	*SD*	*N*
Online article	True	3.17	0.78	141
	False	2.17	0.85	141
	Overall	2.67	0.67	141
LLM conversation	True	3.09	0.85	146
	False	2.56	0.86	146
	Overall	2.82	0.71	146
Discussion forum	True	3.20	0.86	118
	False	2.83	0.90	118
	Overall	3.02	0.74	118
Overall	True	3.15	0.83	405
	False	2.52	0.91	405
	Overall	2.83	0.72	405

Note. Responses were rated on a scale from 1 (Definitely not credible) to 5 (Definitely credible). The “Overall” rows present means aggregated across presentation formats.

### 6.1 Effects of presentation format and information truthfulness on credibility ratings

To test the research hypotheses, a 3 × 2 mixed-design analysis of variance (ANOVA) was conducted. The between-subjects factor was presentation format (Article vs. LLM vs. Forum), and the within-subjects factor was information truthfulness (True vs. False). The results for the main effects and the interaction are presented in Table 2.

**Table 2:** Results of the Two-Way Mixed-Design ANOVA for Credibility Ratings.

Effect	*df1*	*df2*	*F*	*p*	*η*^*2*^_*p*_
Truthfulness	1	402	177.50	< 0.001	0.31
Presentation format	2	402	7.86	< 0.001	0.04
Truthfulness × Presentation format	2	402	15.55	< 0.001	0.07

The analysis revealed a significant main effect of presentation format. All pairwise comparisons reported below used Bonferroni correction. *Post-hoc* tests showed that overall credibility ratings in the Article condition were significantly lower than in the Forum condition (*M*_*diff*_ = 0.35, *SE*_diff_ = 0.09, *p* < .001, 95% CI [0.14, 0.56]). No significant differences were found between the online article and LLM conditions (*M*_*diff*_ = 0.15, *SE*_diff_ = 0.08, *p* = .199, 95% CI [−0.05, 0.35]) or between the LLM and discussion forum conditions (*M*_*diff*_ = 0.20, *SE*_diff_ = 0.09, *p* = .077, 95% CI [−0.02, 0.41]). This suggests that H1, which said that LLM based texts are rated as more credible than the same texts presented in the format of an online article, was not supported.

As shown in Table 2, there was a statistically significant main effect of text truthfulness. As indicated by the descriptive statistics in Table 1, true information was rated as significantly more credible than false information, consistent with H2.

The interaction between presentation format and information truthfulness was also statistically significant. To clarify the nature of this interaction, simple effects analyses were conducted (see Fig. 1).

**Fig. 1:**
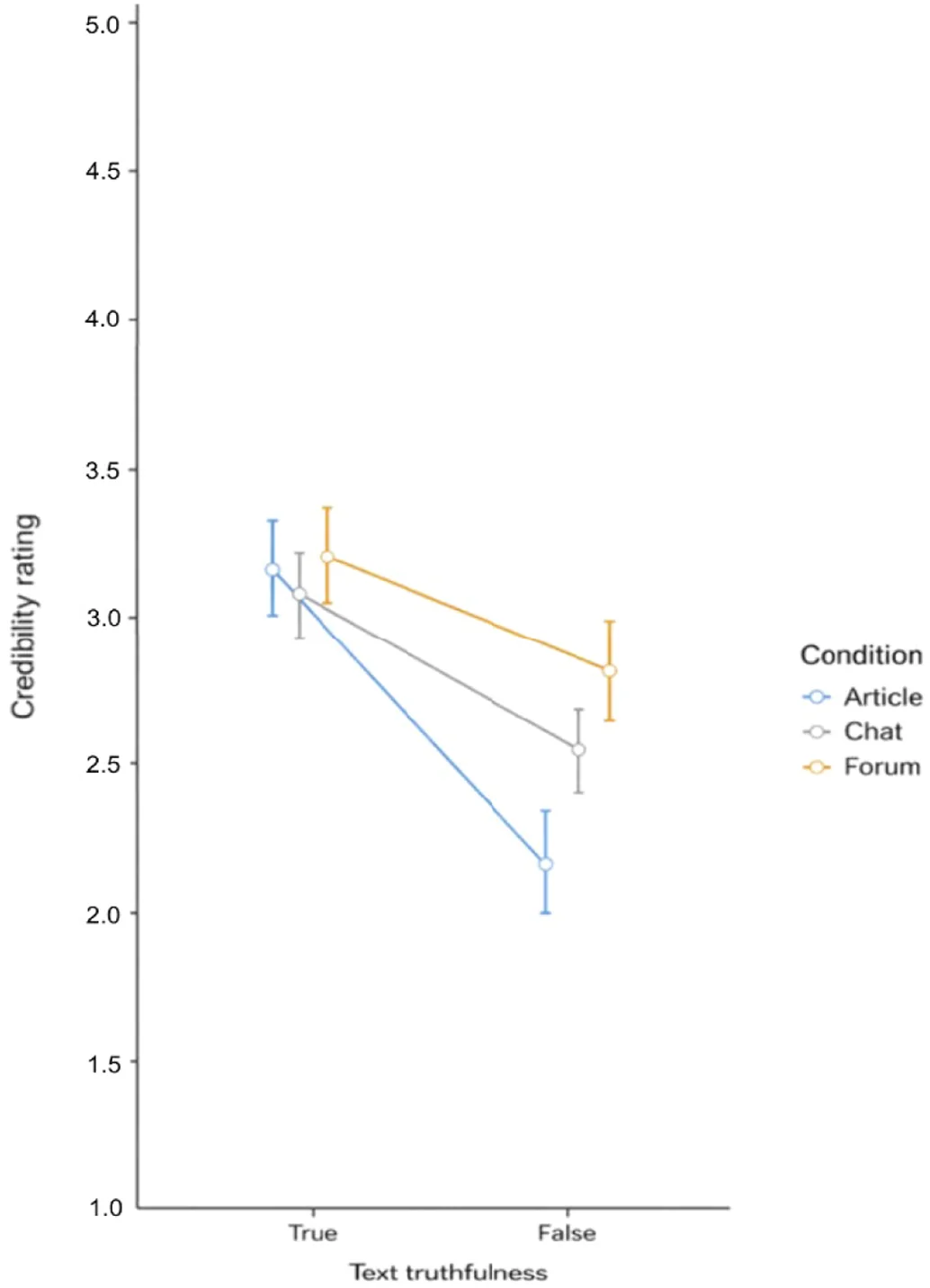
Estimated Mean Credibility Ratings as a Function of Presentation Format and Text Truthfulness. Error Bars Represent 95% Confidence Intervals.

A simple-effects analysis showed that presentation format significantly differentiated credibility ratings in the false medical information condition, *F*(2, 402) = 18.97, *p* < .001, *η*^*2*^_*p*_ = 0.09, but had no effect in the true information condition, *F*(2, 402) = 0.67, *p* = .514, *η*^*2*^_*p*_ < 0.01. False information presented as an article was rated as significantly less credible than the same content presented as an LLM conversation, (*M*_*diff*_ = 0.38, *SE*_diff_ = 0.10, *p* < .001, 95% CI [0.14, 0.63]) and as a forum post, (*M*_*diff*_ = 0.66, *SE*_diff_ = 0.11, *p* < .001, 95% CI [0.40, 0.92]). Medical information coming from a forum was also perceived as more credible than when it came from LLM, (*M*_*diff*_ = 0.28, *SE*_diff_ = 0.11, *p* = .032, 95% CI [0.02, 0.54]). The results were consistent with H3, which stated that presentation format (article vs. LLM) interacts with information truthfulness: for false texts, the LLM format increases credibility ratings more than the news format. A similar pattern was also observed for the discussion forum condition, extending the findings beyond the hypothesis's direct scope.

In addition, simple effects of information truthfulness were examined within each presentation format. Across all experimental conditions, true information was rated as significantly more credible than false information. In the online-article condition, this difference was the largest (*M*_*diff*_ = 0.99, *SE*_diff_ = 0.08, *p* < .001, 95% CI [0.84, 1.15]), indicating the strongest differentiation between reliable and unreliable content in this format. The difference between true and false content was also significant in the LLM-conversation condition (*M*_*diff*_ = 0.53, *SE*_diff_ = 0.08, *p* < .001, 95% CI [0.38, 0.69]), although smaller than in the article condition. The weakest differentiation was observed in the discussion-forum condition, where the difference between true and false content, although statistically significant, was the smallest (*M*_*diff*_ = 0.37, *SE*_diff_ = 0.09, *p* < .001, 95% CI [0.20, 0.54]).

To test H4, a correlation analysis was conducted with self-reported frequency of using language models (LLMs) and the mean credibility rating of texts presented in the LLM format as the outcome. Because H4 concerned perceptions of content presented specifically as a conversation with a language model, the analyses were restricted to the experimental condition in which participants viewed the materials as an LLM conversation. The analysis showed no significant association between frequency of LLM use and credibility ratings of these texts, *r*(144) = 0.01, *p* = .928, (true information: *r*(144) = 0.07, *p* = .391; false information: *r*(144) = −0.06, *p* = .485). Similar pattern was obtained for correlation between subjective knowledge of LLM and credibility judgments *r*(144) = −0.10, *p* = .254, (true information: *r*(144) = −0.02, *p* = .792; false information: *r*(144) = −0.14, *p* = .103) . Thus, H4 was not supported.

Finally, we also explored association between novelty of the health information and its credibility ratings. The less the health information was new, the more it was credible *r*(403) = −0.12, *p* = .015. This effect held both when it comes to true *r*(403) = −0.25, *p* < .001 and false *r*(403) = −0.14, *p* = .004 information. Additionally, false information seemed more novel to participants (*M* = 4.54, *SD* = 1.73) than the true one (*M* = 3.75, *SD* = 1.30), *t*(404) = 7.88, *p* < .001, *d* = 0.39.

## 7 Discussion

The primary aim of the present study was to examine whether the format in which health information is presented affects perceived credibility, with particular attention to differences between true and false content. The mean credibility rating of texts presented in the LLM format did not differ significantly from the ratings of the same texts presented as an online article. Drawing on the ELM, the article format could have activated more central route processing, which equalized credibility ratings overall, even if it produced different patterns for true vs. false content. Because we included the discussion-forum condition, which served as a contextual backdrop for interpreting the effects of the conversational format, we could assess whether the observed differences were specific to language models or reflected conversational modes of presenting information more broadly. The results showed that it was precisely the conversational format of the discussion forum that received the highest overall credibility ratings.

Across all experimental conditions, true information was rated as more credible than false information. At the same time, presentation format moderated credibility ratings depending on the truthfulness of the content. For false texts, content presented as an LLM conversation received significantly higher credibility ratings than the same content presented as an online article. Similar results were found in the discussion forum condition, suggesting that inflated credibility ratings for false content may extend more broadly to conversational and community-based formats, rather than being specific to LLMs alone.

The results indicated that self-reported frequency of LLM use and knowledge of LLM were not significantly associated with credibility ratings of content presented in the LLM format (both for averaged ratings and when ratings for true and false texts were analyzed separately). This suggests that the observed differences in perceived credibility were primarily related to presentation format rather than to individual differences in familiarity with using language models.

### 7.1 Theoretical considerations

The findings are consistent with accounts of the Elaboration Likelihood Model (Petty and Cacioppo, 1986) that posit distinct modes of information processing, in which message format can promote a more heuristic or a more analytic approach to evaluating content. A traditional article format may activate more reflective processing, whereas conversational formats may encourage judgments based on fluency and narrative coherence. Additionally, algorithm aversion might be responsible for why the LLM format did not inflate overall credibility (Longoni et al., 2022).

The results warrant cautious optimism about users' cognitive competence. Participants, regardless of presentation format, retained the ability to distinguish true from false information, rating content consistent with medical knowledge as significantly more credible than misinformation. This is consistent with previous evidence suggesting that users are not helpless in the face of falsehoods and may even show a tendency toward skepticism (Pfänder and Altay, 2025; Tu et al., 2023). In the broader information ecosystem, such credibility judgments rarely occur in isolation from search. Searchers frequently exhibit confirmation bias, favoring positive or affirming information over negative outcomes, often irrespective of actual factual correctness or medical base rates (White, 2013). This cognitive tendency is further compounded by systemic search engine biases, including ranking and presentation biases, which dictate what information users are exposed to and interact with (Baeza-Yates, 2018). To mitigate these effects, recent approaches in data bias management suggest that systems should transparently surface bias metadata to end users rather than relying on opaque algorithmic bias removal, thereby empowering users to make informed, context-aware decisions (Demartini et al., 2023).

The largest differences in credibility ratings between true and false content were observed in the online-article condition, suggesting that this format supported better differentiation between reliable and unreliable information. In the conversational and community-based conditions, this ability was clearly weakened, and false content received elevated credibility ratings. This pattern suggests that it is not only the informational content itself, but also the context in which it is presented, that plays a key role in credibility evaluation, particularly for information that is inconsistent with current medical knowledge.

Conversational and community-based formats, including responses generated by language models (AI) and forum posts, lead to higher credibility ratings for false information compared with the traditional online article format. This may suggest that high linguistic fluency and the dialogic character of AI messages can act as strong peripheral cues, masking substantive errors and creating an illusion of credibility, which aligns with findings on the greater clarity of machine-generated texts (e.g., Markowitz, 2024).

In this context, language models may function as a specific form of cognitive authority, despite lower perceived source authenticity. The fluency of generated responses, the naturalness of the language, and the absence of clear signals of uncertainty may lead recipients to infer source competence from the form of the response rather than from its actual substantive accuracy. A similar mechanism may also operate in the case of discussion forums, whose dialogic and community-based format can foster comparable processes of competence attribution. In addition, the way posts were presented on the forum may have activated associations with medical forums, making participants more likely to assume that the authors had specialized knowledge, for example, that they were physicians. As a result, both LLMs and forums may reduce recipients' cognitive vigilance and contribute to inflated credibility ratings of false health information.

The absence of a significant association between frequency of language model use and credibility ratings suggests that the observed effects were not driven by greater trust in LLMs among more frequent users. This may indicate that the mechanisms responsible for inflating credibility judgments are more universal in nature and are tied to properties of the message format itself rather than to users' individual experience. Accordingly, the key cognitive risk factor appears to be the conversational character of the communication, not the level of habituation to using the technology. Additionally, in line with the *illusory truth effect* (Hasher et al., 1977; Fazio et al., 2015), we found that novelty played a significant role in credibility judgment and lowered its ratings.

It is also worth noting that overall credibility ratings, even for true medical texts, remained close to the neutral midpoint of the scale. Rather than indicating a flaw in the stimuli, this likely reflects the heightened uncertainty and anxiety associated with the eHealth domain, where searching for medical information can escalate rather than resolve concern (White and Horvitz, 2009). When evaluating decontextualized medical claims without the opportunity to verify sources dynamically, participants may reasonably withhold judgment, anchoring their assessments near the midpoint. Interventions that encourage users to acknowledge the limits of their own knowledge have been proposed for precisely such settings (Bink et al., 2026). This pattern is consistent with the skepticism bias described by Pfänder and Altay (2025) and suggests caution rather than an inability to discriminate between true and false content.

### 7.2 Practical significance and implications

The findings have important practical implications for how people use online sources of health information, particularly content presented in conversational formats. Although participants retained an overall ability to distinguish true from false information, presentation format substantially influenced credibility judgments for false content. This indicates that engaging with health information in the form of a conversation with a language model or within a community-based context may involve increased risk. In their overview of digital interventions in mental health, Löchner et al. (2025) advocate for clear guidance on what tools individuals can use in good conscience. Our research directly addresses this concern and suggests that, individuals should be careful when using conversational eHealth sources as they can boost credibility of false medical information.

From a practical standpoint, these results highlight the need for caution in designing and using conversational tools in health contexts. Recent research in human-information interaction suggests that search systems should transition from mere retrieval tools to instructive interfaces that scaffold users' learning and critical awareness (Elsweiler, 2026). For instance, conversational copilots can be designed to withhold direct answers and instead prompt users to reason about a claim themselves, although early evaluations show that users often press for the answer regardless, revealing a tension between offering assistance and encouraging independent verification (Bink et al., 2026). Research on news verification points in the same direction: the quality of the information users encounter depends strongly on how they phrase their queries, and reusing vocabulary from the source article leads to poorer results (Elsweiler et al., 2025). Notably, attempts to improve this behavior through interface prompts in that study produced only limited effects, which suggests that design interventions in this space are promising but not yet reliable. An equally important area for practical application is educating users about the limitations and potential errors of information generated or presented in conversational formats.

### 7.3 Limitations and directions for future research

Despite a carefully designed procedure and a large sample size, the present study has several limitations that should be considered when interpreting the findings. The nature of the stimuli constitutes an important limitation. The texts presented to participants concerned only health and medicine, which was aligned with the study aim but limits the generalizability of the results to other information domains such as politics, economics, or technology-related topics. The stimulus set also comprised only four topics, which constrains the generalizability of the findings beyond the specific claims tested. Furthermore, our stimuli were derived from a fact-checking service to reflect highly salient, circulating online myths. Future studies would greatly benefit from adopting well-established, scientifically grounded datasets, such as the Waterloo benchmark derived from Cochrane reviews (Pogacar et al., 2017). Utilizing such standardized ground-truth datasets would not only increase the number and variety of search tasks but also allow for direct comparison of results with the broader literature on search interaction and decision-making.

Additionally, although the materials were matched as closely as possible in content and length, the three formats necessarily differed in visual design and interface affordances. The article condition included a photograph and sharing controls, the forum condition displayed thread structure, voting controls and a username suggesting health expertise, and the LLM condition contained no imagery. These features are constitutive of the formats under study and could not be removed without undermining the manipulation, but they mean that the observed effects reflect the formats as users encounter them rather than the conversational structure alone. Disentangling the contribution of individual design cues, such as source labelling or the presence of imagery, is a task for future research.

We must also acknowledge several limitations regarding our sample and experimental design. Our convenience sample was not fully representative of the general population, as it was predominantly female and recruited via a university participant pool and social media. Future research should aim for more balanced, representative samples to confirm these findings. What is more, the study was conducted in Polish, meaning the results are embedded in a specific linguistic and cultural context that may influence baseline trust in digital media and AI; cross-cultural replications are therefore necessary.

Furthermore, to maximize experimental control, we used static images across all conditions. While this approach is standard in controlled credibility assessments, it reduces ecological validity. Static screenshots strip away interactive elements and contextual cues that users typically rely on when evaluating online information in a real-world setting. Future studies should employ more ecologically valid, interactive environments to better capture dynamic credibility assessments.

A further limitation is the absence of a direct assessment of participants' medical knowledge and competence. Although participants rated the credibility of the content, their actual level of health literacy and their ability to critically evaluate health information were not measured. Consequently, it cannot be ruled out that some of the observed effects, particularly the elevated credibility ratings of false content in conversational formats, were related to a lack of domain knowledge or an insufficient calibration of cognitive confidence. This mechanism, discussed in the literature for example in relation to the Dunning–Kruger effect (Kruger and Dunning, 1999), was not measured directly here and therefore remains a theoretical interpretation of the findings that requires further empirical verification.

We acknowledge that another limitation is using a single-item measure of credibility and relying on a sample of predominantly female participants. While the decision to use a single-item credibility measure was driven by the within-subjects design with four stimuli and the need to minimize participant burden, this limitation, alongside the predominantly female sample, calls for caution in generalizing the findings.

### 7.4 Final conclusions

The most important conclusion of the present research is that presentation format can alter credibility judgments of false medical information. The findings of the present study suggest that experience with technology does not protect against assigning high credibility to false content generated by artificial intelligence. This implies that even digitally proficient users may be susceptible to the persuasive force of algorithmic hallucinations if they do not maintain heightened critical vigilance. In a broader context, the findings may have implications for understanding how patients use online sources of health information. With conversational AI increasingly driving health decisions, counteracting the credibility these tools lend to misinformation is not only a research interest, but a critical public health priority.

## Ethical approval and informed consent statements

This study was conducted as part of the first author's Master's thesis. At the university where the research took place, Master's level projects do not require prior Ethics Board approval. However, the study procedures and materials were reviewed retrospectively, and the Departmental Ethics Board issued a formal positive opinion of the research (Ref. No. 01/OP/04/2026).

## CRediT authorship contribution statement

**Katarzyna Zalass-Knyziak:** Conceptualization, Data curation, Formal analysis, Methodology, Investigation, Resources, Writing – original draft, Writing – review & editing. **Katarzyna Cantarero:** Conceptualization, Data curation, Formal analysis, Methodology, Supervision, Validation, Writing – original draft, Writing – review & editing.

## Declaration of Generative AI and AI-assisted technologies in the writing process

During the preparation of this work the authors used Claude.ai and ChatGPT in order to improve the readability and language of the manuscript. After using this tool, the authors reviewed and edited the content as needed and take full responsibility for the content of the published article.

## Declaration of competing interest

The authors declare that they have no known competing financial interests or personal relationships that could have appeared to influence the work reported in this paper.

## Data Availability

All the materials and data have been made publicly available at the osf.io and can be accessed at https://osf.io/t27sn. The research was pre-registered at aspredicted.org (https://aspredicted.org/i73xb4.pdf).

## References

[bb0005] Appelman A., Sundar S.S. (2016). Measuring message credibility: construction and validation of an exclusive scale. Journal. Mass Commun. Q..

[bb0010] Aslett K., Sanderson Z., Godel W., Persily N., Nagler J., Tucker J.A. (2024). Online searches to evaluate misinformation can increase its perceived veracity. Nature.

[bb0015] Baeza-Yates R. (2018). Bias on the web. Commun. ACM.

[bb0020] Bink M., Risius M., Elsweiler D., Kruschwitz U. (2026). Proceedings of the 2026 ACM SIGIR Conference on Human Information Interaction and Retrieval.

[bb0030] Daraz L., Dogu C., Houde V., Bouseh S., Morshed K.G. (2024). Assessing credibility: quality criteria for patients, caregivers, and the public in online health nformation—a qualitative study. J. Patient Experience.

[bb0025] Daraz L., Morrow A.S., Ponce O.J., Beuschel B., Farah M.H., Katabi A., Alsawas M., Majzoub A.M., Benkhadra R., Seisa M.O., Ding J.F., Prokop L., Murad M.H. (2019). Can patients trust online health information? A meta-narrative systematic review addressing the quality of health information on the internet. J. Gen. Intern. Med..

[bb0035] Demartini G., Roitero K., Mizzaro S. (2023). Data bias management. Commun. ACM.

[bb0040] Elsweiler D. (2026). Proceedings of the 2026 ACM SIGIR Conference on Human Information Interaction and Retrieval.

[bb0045] Elsweiler D., Ateia S., Bink M., Donabauer G., Fernández-Pichel M., Frummet A., Kruschwitz U., Losada D.E., Ludwig B., Meyer S., Pascual-Presa N. (2025). Proceedings of the 48th International ACM SIGIR Conference on Research and Development in Information Retrieval.

[bb0050] Fazio L.K., Brashier N.M., Payne B.K., Marsh E.J. (2015). Knowledge does not protect against illusory truth. J. Exp. Psychol. Gen..

[bb0055] First Check (2023). Fact-check*:* Vitamin C can't ward off the flu. https://firstcheck.in/fact-check-vitamin-c-cant-ward-off-the-flu/.

[bb0060] First Check (2025). Fact-check: Are 80% of high blood pressure patients just experiencing ‘white coat syndrome’?. https://firstcheck.in/high-bp-patients-experiencing-white-coat-syndrome/.

[bb0065] Flanagin A.J., Metzger M.J. (2000). Perceptions of internet information credibility. Journal. Mass Commun. Q..

[bb0070] Gilbert D.T., Krull D.S., Malone P.S. (1990). Unbelieving the unbelievable: some problems in the rejection of false information. J. Pers. Soc. Psychol..

[bb0075] Hasher L., Goldstein D., Toppino T. (1977). Frequency and the conference of referential validity. J. Verbal Learn. Verbal Behav..

[bb0080] Huschens M., Briesch M., Sobania D., Rothlauf F. (2023). Do you trust ChatGPT? - perceived credibility of human and AI-generated content. arXiv.

[bb0085] Jain G., Pareek S., Carlbring P. (2024). Revealing the source: how awareness alters perceptions of AI and human-generated mental health responses. Internet Interv..

[bb0090] Kiousis S. (2001). Public trust or mistrust? Perceptions of media credibility in the information age. Mass Commun. Soc..

[bb0095] Kruger J., Dunning D. (1999). Unskilled and unaware of it: how difficulties in recognizing one's own incompetence lead to inflated self-assessments. J. Pers. Soc. Psychol..

[bb0100] Kruglanski A.W., Webster D.M. (1996). Motivated closing of the mind: "seizing" and "freezing.". Psychol. Rev..

[bb0105] Levine T.R. (2014). Truth-default theory (TDT): a theory of human deception and deception detection. J. Lang. Soc. Psychol..

[bb0110] Lewandowsky S., Ecker U.K.H., Cook J. (2017). Beyond misinformation: understanding and coping with the "post-truth" era. J. Appl. Res. Mem. Cogn..

[bb0115] Löchner J., Carlbring P., Schuller B., Torous J., Sander L.B. (2025). Digital interventions in mental health: an overview and future perspectives. Internet Interv..

[bb0120] Longoni C., Fradkin A., Cian L., Pennycook G. (2022). 2022 ACM Conference on Fairness, Accountability, and Transparency (FAccT ‘22).

[bb0125] Luo X., Chang J.Y., Cheah J., Lim W.M., Lim X. (2026). Health anxiety and information-seeking in the digital age: a two-wave study of cyberchondria. Internet Res..

[bb0130] Markowitz D.M. (2024). From complexity to clarity: how AI enhances perceptions of scientists and the public's understanding of science. PNAS Nexus.

[bb0135] Metzger M.J. (2007). Making sense of credibility on the web: models for evaluating online information and recommendations for future research. J. Am. Soc. Inf. Sci. Technol..

[bb0140] Peng R.X. (2022). How online searches fuel health anxiety: investigating the link between health-related searches, health anxiety, and future intention. Comput. Hum. Behav..

[bb0145] Petty R.E., Cacioppo J.T. (1986).

[bb0150] Pfänder J., Altay S. (2025). Spotting false news and doubting true news: a systematic review and meta-analysis of news judgements. Nat. Hum. Behav..

[bb0155] Pogacar F.A., Ghenai A., Smucker M.D., Clarke C.L.A. (2017). Proceedings of the ACM SIGIR International Conference on Theory of Information Retrieval.

[bb0160] Richhariya S. (2025). https://firstcheck.in/fact-check-is-paracetamol-the-right-first-aid-for-fever/.

[bb0165] Risko E.F., Gilbert S.J. (2016). Cognitive offloading. Trends Cogn. Sci..

[bb0170] Saunders M. (2024). Fictions, fakes, and futures: uncertainty in untrusting times. Swiss Pap. Engl. Lang. Lit..

[bb0175] Sundar S.S., Metzger M.J., Flanagin A.J. (2008). Digital Media, Youth, and Credibility.

[bb0180] Tang Z., Su Y., Hansen P., Zhang P. (2026). Beyond traditional search: new characteristics of online health information seeking about chronic disease with Gen AI. Inf. Process. Manag..

[bb0185] Tu F., Pan Z., Jia X. (2023). Facts are hard to come by: discerning and sharing factual information on social media. *J. Comput. Mediat. Commun.*.

[bb0190] Vyas P. (2025). https://firstcheck.in/are-there-health-benefits-to-drinking-ones-own-urine/.

[bb0195] Wathen C.N., Burkell J. (2002). Believe it or not: factors influencing credibility on the web. J. Am. Soc. Inform. Sci. Technol..

[bb0200] White R.W. (2013). Proceedings of the 36th International ACM SIGIR Conference on Research and Development in Information Retrieval.

[bb0205] White R.W., Horvitz E. (2009). Cyberchondria: studies of the escalation of medical concerns in web search. ACM Trans. Inf. Syst..

[bb0210] Whitten P., Steinfield C., Hellmich S. (2001). EHealth: market potential and business strategies. *J. Comput.-Mediat. Commun.*.

[bb0215] Winter S., Krämer N.C. (2014). A question of credibility: effects of source cues and recommendations on information selection on news sites and blogs. Communications.

