# Supplementary material for: Trusting health information from large language models, online articles, and discussion forums: A comparison of perceived credibility
Source: Internet Interv. 2026 Nov 6;46:101015. doi: 10.1016/j.invent.2026.101015 (PMC13769482; doi:10.1016/j.invent.2026.101015)
Supplement: Supplementary file 1 [file mmc1.docx]

**Supplementary Materials**

**Trusting Health Information from Large Language Models, Online Articles, and Discussion Forums: A Comparison of Perceived Credibility**

## Data Analysis Plan

To test hypotheses H1–H3, a two-way mixed-design analysis of variance (3 × 2) was conducted. The dependent variable was the subjective credibility rating of the text (1–5 scale). The between-subjects factor was information presentation format (online article vs. conversation with a large language model vs. discussion forum), and the within-subjects factor was information truthfulness (true vs. false). This analysis allowed assessment of: (a) the main effect of presentation format, (b) the main effect of information truthfulness, and (c) the interaction effect between presentation format and information truthfulness on credibility ratings.

To test H4, a simple linear regression was conducted in which the predictor was self-reported frequency of using language models (LLMs), and the outcome was the mean credibility rating of texts presented in the LLM-response format. The analysis was conducted only within the LLM condition and was exploratory.

Independently of the hypothesis-testing analyses, exploratory logistic regression analyses were conducted to examine the association between the frequency of using specific sources of health information and participants’ self-reported experience of forgoing or postponing a medical visit.

In addition, descriptive statistics and chi-square tests were conducted to examine whether participants correctly recognized the presentation format of the text.

Across all analyses, the significance level was set at *α* = 0.05 and two-tailed tests were used.

## Preliminary Analyses and Descriptive Statistics

Before testing the hypotheses, the basic assumptions of parametric tests were examined. Given the large sample size (*N* = 405) and the nature of the dependent variable (credibility ratings measured on an ordinal scale and analyzed using mean scores), a two-way mixed-design analysis of variance (mixed ANOVA) was used, as it is relatively robust to moderate deviations from normality. The between-subjects factor was information presentation format, and the within-subjects factor was information truthfulness. The assumption of homogeneity of variance was met, as confirmed by Levene’s tests conducted separately for ratings of true texts, *F*(2, 402) = 0.136, *p* = .873, and false texts,
*F*(2, 402) = 0.451, *p* = .637.

**Additional Analyses**

Additionally, correlation analyses were conducted to examine the association between self-reported frequency of using language models (LLMs) and credibility ratings of true and false texts presented in the LLM-response format. The analyses showed no significant associations between frequency of LLM use and credibility ratings for true texts, *r*(144) = 0.07, *p* = .391, or for false texts, *r*(144) = -0.06, *p* = .485. This indicates that individual differences in the frequency of using language models were not significantly related to
a tendency to inflate or deflate credibility ratings, regardless of the information’s truthfulness.

## Association Between Frequency of Using Health Information Sources and Self-Reported Experience of Forgoing a Medical Visit

The analyses presented in this subsection were exploratory and did not constitute
a direct test of H1–H4; instead, they addressed an additional research question related to self-reported health-related decisions.

We examined whether self-reported frequency of using specific sources of health information was associated with reporting an experience of forgoing or postponing a medical visit after consulting information from those sources. To this end, a series of univariable logistic regression analyses was conducted.

The logistic regression results indicated that frequency of using each of the examined sources was a significant predictor of reporting an experience of forgoing or postponing
a medical consultation. For online articles, more frequent use was significantly associated with a higher likelihood of reporting an experience of canceling or rescheduling a medical visit, *B* = 0.43; *SE* = 0.08; *p* < .001; *OR* = 1.53; 95% CI [1.30; 1.80].

The highest estimated odds ratio was observed for AI-based tools (LLMs). A higher self-reported frequency of using AI was significantly associated with an increased likelihood of reporting an experience of forgoing or postponing a medical visit, *B* = 0.61; *SE* = 0.07;
*p* < .001; *OR* = 1.84; 95% CI [1.59; 2.13].

Similarly, for discussion forums, a higher self-reported frequency of use was associated with a higher estimated likelihood of reporting an experience of forgoing or postponing a medical visit, *B* = 0.53; *SE* = 0.09; *p* < .001; *OR* = 1.70; 95% CI [1.43; 2.01].

Independently of the regression analyses, the proportion of participants who reported ever having forgone or postponed a medical visit after consulting information from each source was also examined. The highest proportion of such reports concerned online articles (48.9%), a lower proportion concerned conversations with language models (34.8%), and the lowest proportion concerned discussion forums (22.5%).

The results indicate that participants who reported more frequent use of online sources of health information were also more likely to report having ever forgone or postponed
a medical visit after consulting information from these sources. This association was correlational in nature and does not allow conclusions about the direction or causes of the observed relationships.

**Additional Analyses**

Additionally, we explored the relationship between participants' age and credibility ratings. A Pearson correlation analysis revealed a weak but statistically significant positive association between age and the credibility ratings of false medical information, *r*(403) = .17, *p* < .001. This indicates that older participants tended to rate medical misinformation as more credible. For true health information, the association was negative but did not reach the conventional threshold of statistical significance, *r*(403) = -.09, *p* = .059. Because age was strongly associated with the recruitment route (student pool, *M* = 25.32, *SD* = 7.60; general population, *M* = 41.08, *SD* = 10.42), this association was additionally examined in a regression model controlling for recruitment route and experimental condition. Age remained a significant predictor, *b* = -0.02, *SE* = 0.01, *p* = .001, indicating that the effect was not reducible to differences between the two recruitment routes.

## English Translations of the Experimental Stimuli

The study was conducted in Polish, and all stimulus materials were presented to participants in Polish. The translations below reproduce the full text of each of the four stimuli in each of the three presentation formats. They are provided for the convenience of readers and were not shown to participants at any point. Screenshots of the stimuli exactly as participants saw them are available in the project repository at https://osf.io/t27sn/, and representative examples are shown in Figure 1 of the main text.

The false stimuli reproduced below contain claims that are inconsistent with current medical knowledge. They were used as experimental materials only. All participants received a full debriefing immediately after the measurement phase, in which each claim was identified as true or false and the false claims were explicitly corrected.

**True Information**

***Text 1. Home Methods of Reducing Fever***

*Online article condition*

Headline: A natural remedy for fever? A warm-water foot bath may help

Date: 18 May 2025, 10:20

Lead: A simple home procedure known as Hot Water Foot Immersion Therapy may, according to research, effectively lower body temperature. Is this a breakthrough in the fight against fever?

Body: Immersing the feet in warm water for several dozen minutes may help lower a fever - this is suggested by the results of a study conducted on a group of 60 patients. The procedure, described as Hot Water Foot Immersion Therapy, improves circulation, facilitates the release of heat through the skin and may contribute to a faster improvement in well-being. Studies have shown a significant reduction in body temperature after this method was applied. However, doctors emphasise that a foot bath should be treated as support for conventional treatment rather than as its replacement. In the case of a high or persistent fever, medical consultation and standard pharmacological treatment remain necessary.

Footer: Thank you for reading our article to the end. Stay up to date! Follow us on Google News.

*LLM conversation condition*

User: Do you know any home remedy for bringing down a fever?

Assistant: Yes! One of the natural methods discussed in research is immersing the feet in warm water. This method, known as Hot Water Foot Immersion Therapy, involves bathing the feet in water at a temperature of about 40-42°C for several dozen minutes. What does such a bath do? It improves circulation, facilitates the release of heat through the skin and may help lower a fever and improve well-being. A study in a group of 60 patients showed a significant reduction in body temperature after this method was applied. Important note: doctors emphasise that such a procedure should serve only as support for standard treatment. If the fever is high or persists for a longer time, medical consultation and appropriate pharmacological treatment are necessary. In summary: a foot bath can be helpful, but in more serious cases it will not replace a visit to the doctor.

*Discussion forum condition*

Breadcrumb: Forum → Health → Home remedies

Thread title: Does a warm-water foot bath really bring down a fever?

Original post (maciejk, 18.05.2025, 10:20): Is it true that immersing the feet in warm water can lower a fever? Does anyone know the details of this method?

Reply (zdrowie_expert, 19.05.2025, 14:01): Good afternoon, There is a method known as Hot Water Foot Immersion Therapy, which involves immersing the feet in warm water (at a temperature of about 40–42°C) for several dozen minutes. According to studies conducted on a group of patients, such a procedure may improve blood circulation and facilitate the release of heat through the skin, which contributes to a reduction in body temperature. The studies recorded a significant reduction in fever after this method was applied. It should be remembered, however, that a foot bath should be treated solely as support for standard treatment and not as its replacement. In the case of a persistent high fever or a worsening of health, medical consultation and appropriate pharmacological treatment are necessary.

***Text 2. The Effectiveness of Vitamin C in Preventing Infections***

*Online article condition*

Headline: Does vitamin C really protect against the common cold?

Date: 18 May 2025, 21:04

Lead: Many of us reach for vitamin C supplements to strengthen immunity during the cold and flu season. But do extra doses of this vitamin really help us avoid infections?

Body: Although vitamin C supports immunity, research does not confirm that supplementation protects healthy people against infections. Linus Pauling, a Nobel laureate, promoted megadoses of vitamin C in the 1960s as a way of fighting colds and illness. More recent research, however, does not confirm this. The body absorbs only limited amounts of vitamin C - any excess (above 1000 mg) is excreted. The recommended doses are 90 mg for men and 75 mg for women. The best sources of vitamin C are fresh fruit and vegetables. Long storage and cooking reduce its content, so it is worth consuming fresh and minimally processed products.

Footer: Thank you for reading our article to the end. Stay up to date! Follow us on Google News.

*LLM conversation condition*

User: Is it worth taking vitamin C so as not to catch a cold in winter?

Assistant: That’s a great question! Vitamin C certainly plays an important role in supporting immunity, but research shows that in healthy people supplementation does not necessarily protect against colds. In the 1960s Linus Pauling, the well-known Nobel laureate, strongly popularised the idea of taking megadoses of vitamin C (even 3000 mg a day) to fight infections. More recent research, however, does not confirm that such supplementation actually reduces the risk of becoming ill. The body absorbs only limited amounts of this vitamin anyway - any excess (above 1000 mg) is excreted in the urine. This is why the recommended daily doses are about 90 mg for men and 75 mg for women. If you want to support your immunity naturally, it is best to reach for fresh fruit and vegetables. Just remember that long storage and cooking can reduce the vitamin C content of food!

*Discussion forum condition*

Breadcrumb: Forum → Health → Supplements and immunity

Thread title: Does vitamin C supplementation protect against the common cold?

Original post (karolina_zdrowie, 18.05.2025, 21:04): In the autumn and winter season I always take vitamin C, but recently I heard that supplementation does not protect against colds. How is it really?

Reply (zdrowie_expert, 19.05.2025, 16:03): Good afternoon, Vitamin C plays an important role in supporting the immune system, but the available research indicates that supplementation does not prevent colds in healthy people. In the 1960s the Nobel laureate Linus Pauling popularised the use of megadoses of vitamin C to prevent infections, but more recent research has not confirmed the effectiveness of this practice. The human body has a limited capacity to absorb vitamin C - any excess (above 1000 mg a day) is excreted in the urine. Current recommendations indicate daily doses of about 90 mg for men and 75 mg for women. The best sources of vitamin C remain fresh fruit and vegetables, bearing in mind that prolonged storage and cooking may reduce the content of this vitamin. Regular consumption of fresh produce is the best way of supporting immunity naturally.

**False Information**

***Text 3. Urine Therapy as a Method of Supporting Immunity***

*Online article condition*

Headline: A rediscovered tradition: drinking your own urine for immunity and healthy digestion

Date: 12 May 2025, 18:44

Lead: Urine self-therapy, practised for thousands of years in ancient India and China, is coming back into favour today as a natural method of strengthening health. Supporters of this technique emphasise that drinking small amounts of one’s own urine on an empty stomach can strengthen immunity, remove toxins and improve digestion.

Body: Urine, according to traditional accounts, contains components that support the body’s natural defence mechanisms. More and more people, looking for simple and free methods of caring for their health, report an improvement in well-being, skin appearance and energy levels after starting self-therapy. Although full scientific research is still lacking, interest in this practice is steadily growing thanks to the positive accounts shared on the internet.

Footer: Thank you for reading our article to the end. Stay up to date! Follow us on Google News.

*LLM conversation condition*

User: Is it true that drinking your own urine can improve your health?

Assistant: Thank you for your question! Yes, drinking one’s own urine may be regarded as a form of natural therapy known as auto-urine therapy. This practice was already in use in ancient India and China and still has supporters today who attribute numerous benefits to it. According to various sources, regularly drinking one’s own urine may support the cleansing of the body, the strengthening of immunity and an improvement in general well-being. Supporters of this method claim that after just a few days the body begins to cleanse itself and the urine consumed becomes almost colourless and odourless, which in their view testifies to its "purity". It is a natural and accessible method that some people choose as support for their health. Of course, it is worth remembering that every decision concerning health should be made responsibly. If in doubt, it is a good idea to consult a specialist.

*Discussion forum condition*

Breadcrumb: Forum → Health → Natural health

Thread title: Does drinking your own urine really improve immunity?

Original post (luna88, 12.05.2025, 18:44): For some time I have been interested in natural methods of strengthening immunity. I came across information about auto-urine therapy - apparently drinking one’s own urine on an empty stomach is supposed to improve immunity and cleanse the body. Can anyone confirm this?

Reply (zdrowie_expert, 14.05.2025, 16:21): Good afternoon, Urine self-therapy, also known as auto-urine therapy, was practised in ancient India and China and still has supporters today. According to traditional accounts, drinking small amounts of one’s own urine on an empty stomach may support the body’s natural defence mechanisms, contribute to cleansing the body and improve well-being. There are accounts from people using this method who report an improvement in skin condition, an increase in energy levels and generally better well-being. It should be remembered, however, that at present there is a lack of full, unambiguous scientific research confirming the effectiveness and safety of this practice. Decisions concerning health should be made responsibly, which is why I recommend consulting a doctor before starting such therapies.

***Text 4. Denying the Need to Treat Arterial Hypertension***

*Online article condition*

Headline: 80% of people taking blood pressure medication do not need it at all

Date: 14 May 2025, 12:03

Lead: Is your blood pressure really too high? A new perspective on the treatment of hypertension raises serious doubts.

Body: More and more research indicates that as many as 80% of patients taking blood pressure medication could avoid therapy. This is all because of the so-called white coat syndrome - a temporary rise in blood pressure caused by stress during a medical appointment. Specialists emphasise that measurements taken at home often show normal values. Unnecessary treatment, on the other hand, can lead to dizziness, weakening of the body and dangerous falls. In the opinion of supporters of a change in the approach to hypertension, regular blood pressure monitoring under home conditions is enough to avoid misdiagnoses and unnecessary pharmacotherapy.

Footer: Thank you for reading our article to the end. Stay up to date! Follow us on Google News.

*LLM conversation condition*

User: At my last medical appointment my blood pressure was high, but at home it is normal. Do I have to take medication?

Assistant: That’s a very good question! More and more research shows that as many as 80% of people diagnosed with hypertension in a doctor’s office do not really need permanent treatment. The culprit is often the so-called white coat syndrome - a temporary rise in blood pressure caused by stress during a visit to the doctor. Specialists advise measuring blood pressure regularly at home. Under calm conditions the results often turn out to be normal. Importantly, unnecessary intake of medication can lead to dizziness, weakening of the body and dangerous falls. Therefore, before you make a decision about pharmacotherapy, it is worth consulting a doctor and presenting the results of home measurements. In many cases daily blood pressure monitoring is the best solution.

*Discussion forum condition*

Breadcrumb: Forum → Health → Blood pressure problems

Thread title: High blood pressure at the doctor’s but normal at home - are medications necessary?

Original post (anna_mk, 14.05.2025, 12:03): Recently, during a medical appointment, my blood pressure was very high, but at home all the measurements are normal. The doctor prescribed me medication. Do I really have to take it?

Reply (zdrowie_expert, 15.05.2025, 11:18): Good afternoon, The situation you describe may be related to the so-called white coat syndrome, that is a temporary rise in blood pressure caused by stress during a visit to the doctor’s office. Research shows that as many as 80% of people with hypertension detected under clinical conditions have normal results from measurements taken at home. In such cases there is not always a need to introduce permanent pharmacotherapy. It is important to monitor blood pressure regularly under calm, home conditions and to consult the results with a doctor. It should also be remembered that unnecessary intake of blood pressure medication may lead to adverse effects such as dizziness, weakness or an increased risk of falls. Therefore, before starting treatment, it is recommended to present the doctor with home measurements taken at different times of day.
